# Metastatic melanoma patients’ sensitivity to ipilimumab cannot be predicted by tumor characteristics

**DOI:** 10.1097/IJ9.0000000000000043

**Published:** 2017-10-10

**Authors:** Kara Rossfeld, Erinn M. Hade, Alexandra Gangi, Matthew Perez, Emily N. Kinsey, Joanna Grabska, Ashley Ederle, Jonathan Zager, April K. Salama, Thomas E Olencki, Georgia M Beasley

**Affiliations:** a Division of Surgical Oncology; b Department of Biomedical Informatics, Center for Biostatistics, The Ohio State University College of Medicine, Columbus, OH; c H. Lee Moffitt Cancer Center, Tampa, FL; d Division of Medical Oncology, Duke University, Durham, NC; e Department of Internal Medicine, Division of Medical Oncology, College of Medicine, The Ohio State University Wexner Medical Center, Columbus, OH; f Division of Advanced Oncologic and Gastrointestinal Surgery, Duke University, Durham, NC

**Keywords:** Melanoma, Ulceration, BRAF, Ipilimumab

## Abstract

Immune checkpoint inhibitors have dramatically changed the prognosis for patients with metastatic melanoma. However, not all patients respond to therapy and toxicities can be severe leaving need for reliable clinical predictive markers.

**Methods:**

We examined primary tumor characteristics including ulceration, BRAF mutation status, and Breslow depth in patients who subsequently developed stage IV disease and were treated with ipilimumab at 3 institutions. Patients in this study were not treated on clinical trials. To investigate the relationship between patient characteristics at the time of diagnosis and survival following melanoma diagnosis we utilized Cox proportional hazards models, accounting for delayed entry into the study cohort. Cox models estimate the age and institution adjusted hazard ratios for risk of death.

**Results:**

Of patients (n=385) treated with ipilimumab for stage IV melanoma, 302 met inclusion criteria. The complete response to ipilimumab was 5%, partial response was 13%, 18% had stable disease, 62% had progressive disease, and 5 unknown. The median overall survival rate was 2.03 years [95% confidence interval (CI): 0.13, 3.05]. Primary tumor Breslow depth, lymphovascular invasion, BRAF status, and ulceration did not predict sensitivity to ipilimumab. In this study patient cohort, BRAF mutation (adjusted hazard ratio: 1.43, 95% CI: 0.98, 2.07) and presence of ulceration (adjusted hazard ratio: 1.47, 95% CI: 0.95, 2.26) contributed to an increased risk of death.

**Conclusions:**

The presence of ulceration did not correlate with sensitivity to ipilimumab. Ulceration of the primary tumor and a BRAF mutation were moderately associated with worse survival in patients with metastatic melanoma treated with ipilimumab.

## Background

The median survival for patients with distant melanoma metastases has historically been <1 year^1^. The rapid emergence of novel therapeutics in advanced melanoma, including immune checkpoint inhibitors such as ipilimumab and Programmed cell death protein 1 inhibitors has resulted in improved overall survival (OS)^2^,^3^. Ipilimumab, a monoclonal antibody directed against the CTLA-4 protein, promotes immune recognition of self-antigens and thus unleashes cytotoxic activity of endogenous melanoma antigen-specific T lymphocytes^2^. Ipilimumab and Programmed cell death protein 1 inhibitors have been shown to improve OS in patients with metastatic melanoma^2^,^3^. However, side effects including immune-related adverse events are common with 10%–15% of patients treated with ipilimumab developing severe, grade 3 or higher toxicities^2^. Therefore, appropriate patient selection is critical to maximize response while minimizing toxicity.

Previous studies have examined clinical and biologic predictors of response to ipilimumab including serum immunoregulatory proteins, lactate dehydrogenase (LDH) levels, and BRAF or NRAS mutational status^4^–^6^. Response to immune checkpoint inhibitors seems to correlate with higher mutational load, though studies have been inconsistent^7^,^8^. Melanoma is both a highly mutated and highly immunogenic tumor as evidenced by lymphocyte responses to primary melanoma and melanoma differentiation antigens^9^. This immunogenicity is thought to be related in part to the high mutational load in melanoma^7^. Ulceration of primary cutaneous melanoma is a known adverse prognostic factor and represents highly undifferentiated disease^9^. Meta-analysis of 15 adjuvant trials of adjuvant interferon (IFN)-alpha and long-term follow-up of the EORTC 18952 trial of adjuvant IFN found ulceration of the primary melanoma was the key determinant for IFN sensitivity^10^,^11^. Furthermore, the presence of tumor infiltrating lymphocytes (TILs) and tumor infiltrating dendritic cells has previously been shown to be associated with prognosis and response to historic (IFN) immunomodulatory therapies^12^. To our knowledge, no other studies have specifically examined the association of primary tumor ulceration or presence of TILs and response to ipilimumab in the metastatic setting.

Interestingly, a randomized phase 3 trial of ipilimumab versus placebo in stage III resected melanoma initially showed that patients with ulcerated melanoma appeared to benefit more from ipilimumab than patients with nonulcerated primary with a hazard ratio of 0.67 (0.48–0.93) for survival in favor of ipilimumab treatment^13^. However, long-term follow-up of that study showed adjuvant ipilimumab prolonged survival compared with placebo in patients with both ulcerated and nonulcerated melanomas^14^. Thus, it is unclear if ulceration as a marker of dedifferentiation and subsequent immunogenicity is an important prognosticator for immune blockade therapy. The aim of this study is to examine the relationship between primary tumor characteristics and survival in patients who developed metastatic disease and were treated with ipilimumab.

## Methods

Patients with metastatic melanoma of cutaneous origin and a known primary site who were treated with ipilimumab at the Ohio State University Wexner Medical Center (OSU), Duke University and H. Lee Moffitt Cancer Center (Moffitt) were identified. Patients on clinical trials, those younger than 18 years at the time of metastatic disease, and those with mucosal and uveal melanoma patients were excluded. Patients who did not have recorded BRAF mutation status were also excluded. Treatment with ipilimumab was defined as receiving at least 1 dose (3 mg/kg). Pathology reports from the primary melanoma were reviewed and the following factors recorded: ulceration, Breslow depth, TILs (absent or present), and the presence of BRAF V600E mutation. Response was defined in this study according to immune-related response criteria^15^. Summary statistics were performed using percentages and proportions where appropriate.

To investigate the relationship between patient characteristics at the time of diagnosis and survival following melanoma diagnosis, we utilized Cox proportional hazards models. Patients entered the study cohort at the time of treatment, but are at risk of death from disease from the time of diagnosis until the time of last follow-up. Left truncation (delayed entry into this cohort) is accounted for by including the age at treatment with ipilimumab into the at-risk time calculation. Cox models estimate the age and institution adjusted hazard ratios for risk of death, age is used as the time scale, and the baseline hazard is stratified by institution in all analyses. All reported *P*-values are 2-sided and unadjusted for multiple comparisons. Analyses and data management were conducted in Stata 13.0 (StataCorp, 2013). The institutional review boards at the corresponding institutions approved this study.

## Results

From 2012 to 2015, a total of 385 patients with metastatic melanoma received ipilimumab at the 3 centers. A total of 83 patients were excluded including 3 patients with metastatic mucosal melanoma, 62 patients with metastatic melanoma of unknown primary, 1 patient younger than 18 at the time of treatment, and 18 with no known BRAF mutation status. The final analysis cohort included 302 adults with metastatic melanoma including 73 from OSU, 119 from Duke, and 110 from Moffitt.

Patient characteristics are described in **Table 1**. Twenty-eight (9%) patients had stage I disease, 35% (n=106) had stage II disease, 46% (n=139) had stage III disease, 7% (n=22) had stage IV disease at initial melanoma diagnosis, and initial stage was unknown in 8 patients. The median time from initial diagnosis to diagnosis of stage IV disease was 18.3 months. At time of treatment in 302 patients in whom data was known, 50 (17%) had stage M1a disease, 61 (20%) had M1b disease, and 175 (58%) had M1c disease. At time of treatment, the mean LDH was 464 U/L (n=104). 62% (189/302) of patients had received prior systemic therapies including high-dose interleukin-2, interferon-alfa 2b, sargramostim, temozolomide, and vemurafenib. Of the 302 patients treated, 75% (n=226) received 4 doses of ipilimumab. In the 76 patients in whom the complete course was not given, 34 patients (46%) stopped due to toxicity, 15 patients (20%) stopped due to progression of disease, and unclear from data in the remaining 27 (34%) patients. Overall, 71.5% (n=216) patients reported complications of any grade, specific grades of toxicity were not recorded.

**Table 1:**
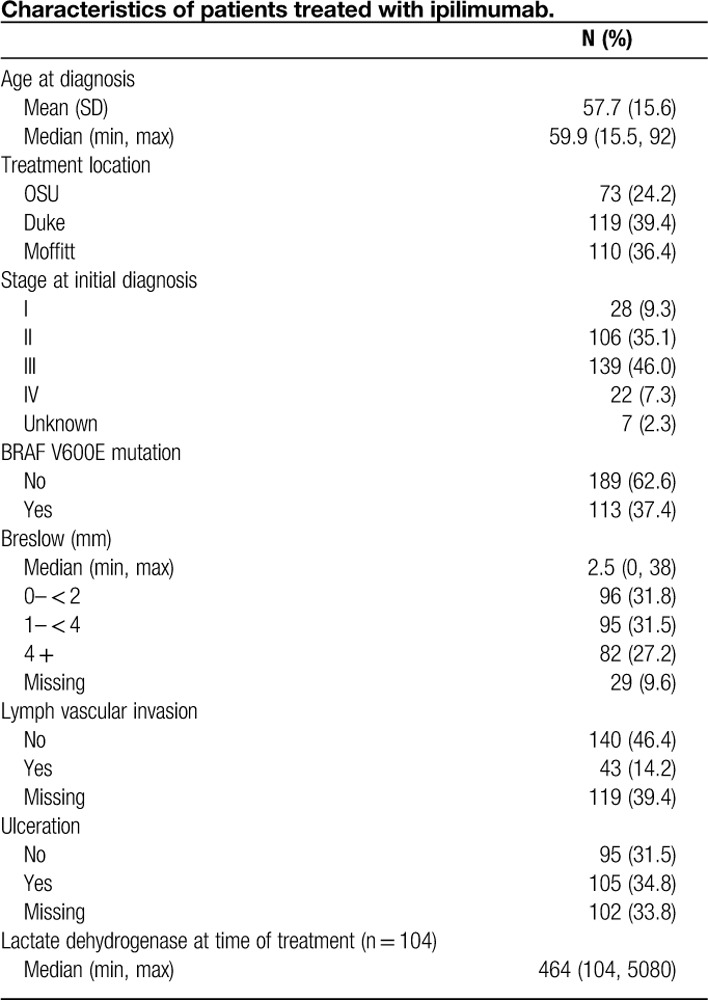
Characteristics of patients treated with ipilimumab.

Tumor characteristics are described in **Table 2**. The median Breslow depth was 2.5 mm, 52% (n=105/200) of patients had ulcerated primary lesions, 77% (143/186) had TILs present, and 37% (n=113/302) were BRAF mutation positive (specific V600 subtypes were not collected). The complete response rate to ipilimumab was 5% [15/302, 95% confidence interval (CI): 0.03, 0.08], and the partial response was 13% (39/302, 95% CI: 0.09, 0.17). Fifty-six patients (18%) had stable disease, 187 patients (62%) had progressive disease, and response was unknown in 5 patients. There was no difference in primary tumor Breslow depth, presence or absence of TILs, *BRAF* status, and ulceration status between responders and nonresponders. Specifically in patients with data available, there was no significant difference in the presence of ulceration between responders (31%, 17/54) and nonresponders (36%, 88/243). Similarly, there was no difference in the presence of TILs between responders (43% 23/54) and nonresponders (49%, 120/243).

**Table 2:**
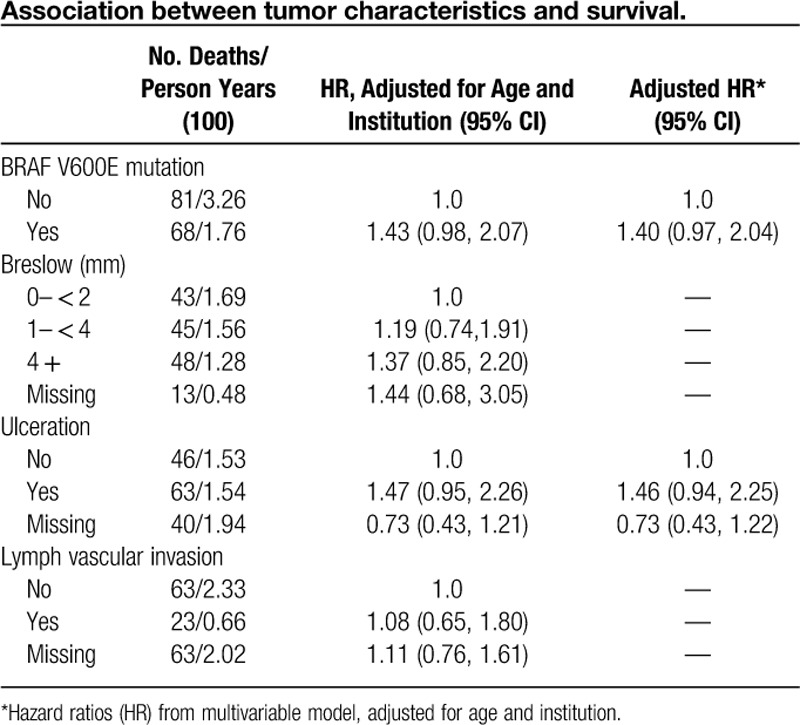
Association between tumor characteristics and survival.

**Figure 1:**
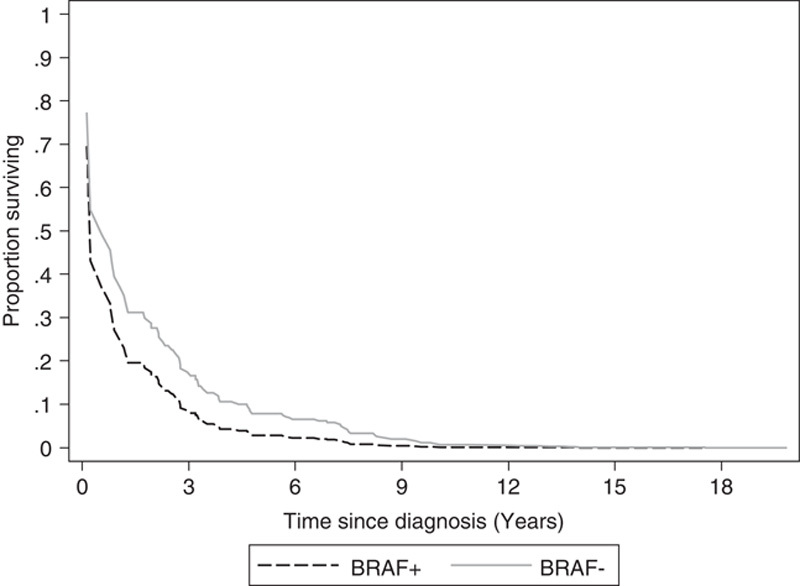
Adjusted overall survival curve comparing BRAF-positive and BRAF-negative patients. Survival function is estimated from the adjusted Cox proportional hazards model for those with ulceration and an average age of 50; baseline hazard is stratified by the institution. Figure is for 1 institution.

During the follow-up period, 149 deaths were observed over a median follow-up of 3.95 years (range, 0.13–32.0 y). The median OS rate was 2.03 years (95% CI: 0.13, 3.05), with 5-year survival probability of 22% (95% CI: 14.2%, 31.6%). **Table 2** describes associations between each tumor characteristic and survival, adjusted for patient age at diagnosis and institution. BRAF mutation (adjusted hazard ratio (aHR): 1.43, 95% CI: 0.98, 2.07) and presence of ulceration (aHR: 1.47, 95% CI: 0.95, 2.26) at the time of diagnosis were both marginally associated with OS, individually. When adjusted for one another, their estimated effect on survival remained similar (BRAF aHR: 1.40, 95% CI: 0.97, 2.04; ulceration aHR: 1.46, 95% CI: 0.94, 2.25) suggesting that each contributed to an increased risk of death. **Figure 1** depicts the estimated adjusted survival curve comparing patients who were BRAF positive to those BRAF negative with ulceration, with an average age of 50 (presented for 1 institution).

## Discussion

As with any therapy, optimal patient selection to maximize response while minimizing risk is critical. Immune checkpoint inhibitors have shown efficacy in metastatic melanoma but determination of which patients are most likely to derive benefit in advance of therapy remains unclear. Without robust predictors of response, patients may be unnecessarily exposed to toxicities without an expected benefit. There are currently no cost effective, minimally invasive widely utilized methods of predicting response to ipilimumab. In the present study, primary tumor characteristics including presence of ulceration were not found to be associated with OS benefit from ipilimumab.

Ulceration is a strongly proven predictive marker for sensitivity to adjuvant IFN^10^,^11^,^16^–^19^. In EORTC 18071, which examined adjuvant ipilimumab after adequate resection of stage III cutaneous melanoma, initial results showed that patients with ulcerated melanoma appeared to benefit more from ipilimumab than patients with nonulcerated primaries with a hazard ratio of 0.67 (0.48–0.93) for survival in favor of ipilimumab treatment^13^. Final results showed adjuvant ipilimumab increased the 3-year recurrence-free survival rate (46.5% for ipilimumab versus 34% placebo) (*P*=0.0013) with no differences in subgroup analysis between patients with or without ulceration^14^. These results and our study suggest the relationship between ulceration and response to historic immune therapy may not be applicable to modern immune blockade therapy leaving a need to search for simplified means of determining candidates most appropriate for ipilimumab^14^. Other clinical factors like the presence of serum immunoregulatory proteins, prognostic scores based on serum LDH, and BRAF/NRAS mutation status^4^–^6^ have been examined with no conclusive results^4^–^6^,^20^.

Using clinical factors like primary tumor characteristics to predict response to immune therapy may not be helpful in patients with metastatic disease because the important molecular and genetic changes known to occur during progression of cancer from localized disease to distantly metastatic are not included. It is possible that these changes are equally or even more important than primary tumor characteristics as far as their impact on tumor response to checkpoint inhibitor therapy. Sophisticated studies have found a genetic basis to clinical benefit from CTLA-4 blockade including overall mutational load, neoantigen load, and expression of cytolytic markers in the immune microenvironment^7^,^19^. In addition, changes in absolute lymphocyte counts and T-cell subsets may be predictive after initiating therapy but patients may still be exposed to therapy unnecessarily with this method^21^. Ultimately, prediction of response to immune blockade may require more complex models.

Our study is limited by the retrospective nature and we also examined a heavily pretreated population (62% had prior systemic therapy), which could have impacted our findings. Our population does reflect a more broad experience of patients not on clinical trials and importantly rates of therapy completion, response, and survival are similar to those reported in clinical trials^2^. Specifically, the best overall response rate in 137 patients treated with ipilimumab at a dose of 3 mg/kg every 3 weeks was 10.9% with complete response being 1.5% in a large randomized trial^2^. Interestingly, in our study, only 75% (n=223/302) completed 4 cycles of ipilimumab similar to rates of 60%–64% in a large randomized trial^2^. Because trials utilize intention to treat analysis, the impact of not completing 4 cycles of therapy is not well understood.

A final interesting study finding was that the presence of a *BRAF* mutation had a moderate increased risk of death while adjusting for ulceration, age, and institution in these patients (n=302) with metastatic melanoma. The impact of *BRAF* mutation on prognosis in melanoma is currently still being investigated. The presence of a *BRAF* mutation was also found to be strongly associated with inferior survival in the metastatic setting in a previous study of 45 patients^22^. However in that same small study, *BRAF* status did not influence disease-free interval from diagnosis to metastases^22^. In addition, another study showed no difference in OS according to *BRAF* status^23^. Finally, a population-based study in 912 patients with primary cutaneous melanoma from the United States and Australia found that melanoma-specific survival was significantly poorer for higher risk (T2b or higher) tumors with a *BRAF* (HR=3.1, 95% CI 1.2–8.5) mutation but not for lower risk tumors^24^. High-risk tumors are more likely to become metastatic and thus again this may suggest that the presence of *BRAF* in the metastatic setting confers a worse prognosis. In addition, if *BRAF* status is associated with worse OS, whether or not this can be overcome with *BRAF*-inhibitor therapy is unknown.

## Conclusions

The presence of ulceration in patients’ primary tumors that subsequently develop metastatic melanoma did not seem to have an association with sensitivity to treatment with ipilimumab. In addition other routine primary tumor characteristics do not seem to predict sensitivity to ipilimumab and more complex approaches may be needed to identify responders before initiation of therapy to avoid unnecessary toxicities.

## Ethical approval

Institutional Review Boards at the corresponding institutions approved this study.

## Sources of funding

Supported by The Ohio State University Comprehensive Cancer Center (National Cancer Institute Grant P30 CA016058).

## Author contribution

All authors read and approved the manuscript.

## Conflict of interest disclosures

T.E.O.: has clinical trial support from Pfizer, Bristol Myers Squibb, and Tracon, T.E.O. serves on the advisory board for Genetech but received no financial benefits. A.S.: has Research funding (paid to institution): Bristol Myers Squibb, Celldex, Genentech, Immunocore, Reata. A.S. is a consultant for Bristol Myers Squibb. The remaining authors declare that they have no financial conflict of interest with regard to the content of this report.

## Research registration unique identifying number (UIN)

Not applicable.

## Guarantor

Not applicable.

## Acknowledgments

The authors are indebted to all the members who helped them complete this study.
